# Performance of Seven Tree Breeding Strategies Under Conditions of Inbreeding Depression

**DOI:** 10.1534/g3.115.025767

**Published:** 2016-01-04

**Authors:** Harry X. Wu, Henrik R. Hallingbäck, Leopoldo Sánchez

**Affiliations:** * Umeå Plant Science Centre (UPSC), Department of Forest Genetics and Plant Physiology, Swedish University of Agricultural Sciences, 901 87 Umeå, Sweden; † Commonwealth Scientific and Industry Research Organization (CSIRO), Black Mountain Laboratories, Canberra, ACT 2601, Australia; ‡ Institut National de la Recherche Agronomique (INRA)-Orleans, Unité d’Amélioration, Génétique et Physiologie des Arbres Forestiers, B. P. 20619, 45166 Olivet Cedex, France

**Keywords:** breeding strategy, finite locus model, multiple population, subline, nucleus, inbred-hybrid, genetic gain

## Abstract

In the domestication and breeding of tree species that suffer from inbreeding depression (ID), the long-term performance of different breeding strategies is poorly known. Therefore, seven tree breeding strategies including single population, subline, selfing, and nucleus breeding were simulated using a multi-locus model with additive, partial, and complete dominance allele effects, and with intermediate, U-shaped, and major allele distributions. The strategies were compared for genetic gain, inbreeding accumulation, capacity to show ID, the frequencies and fixations of unfavorable alleles, and genetic variances in breeding and production populations. Measured by genetic gain of production population, the nucleus breeding and the single breeding population with mass selection strategies were equal or superior to subline and single breeding population with within-family selection strategies in all simulated scenarios, in spite of their higher inbreeding coefficients. Inbreeding and cross-breeding effectively decreased ID and could in some scenarios produce genetic gains during the first few generations. However, in all scenarios, considerable fixation of unfavorable alleles rendered the purging performance of selfing and cross-breeding strategies ineffective, and resulted in substantial inferiority in comparison to the other strategies in the long-term.

Most tree breeding programs in the world were initiated in the 1950s with plus tree selection and progeny testing (Zobel and Talbert 1984), and many of them have now entered into the second, third, or even fourth generation (*e.g.*, loblolly and radiata pine). Recurrent selection has been the principal method for the improvement of tree species (White 2001; White *et al.* 2007). In order to increase short- and long-term genetic gains and to manage inbreeding and diversity, several advanced tree breeding strategies were proposed including multiple populations (Namkoong 1976), sublines (van Buijtenen and Lowe 1979), nucleus breeding (Cotterill 1989), single population breeding, and inbred-hybrid (Wu *et al.* 2004a). In this context, inbreeding management is important because a too rapid increase in inbreeding and concomitant decrease in heterozygosity has been shown to cause severe inbreeding depression (ID) in several conifer species (Williams and Savolainen 1996). The prevalence of alleles exhibiting dominant gene action (complete, partial or overdominance) and directionality (most recessives being unfavorable) has been proposed as the main mechanism for causing ID and its opposite counterpart hybrid vigor (Falconer and Mackay 1996). The symptoms of ID in conifers usually include reduced seed production, impaired growth, and decreased adult fecundity.

Breeding in a single population with recurrent selection for general combining ability has been the default option that was initially used for many tree breeding programs (Shelbourne 1969). To increase genetic gain and selection intensity, this strategy has evolved into a rolling-front mating and selection design where the breeding workload is spread within a virtual breeding cycle by continuous crossing, testing, and selection each year (Borralho and Dutkowski 1998). The greatest strength of the single population scheme is the increased selection intensity made possible by its potentially large size, together with the ease of limiting inbreeding by mating selection managing tools (Kinghorn 2011).

However, inbreeding can also be managed by a subline strategy where the breeding population is subdivided into two or multiple groups (sublines, Burdon and Namkoong 1983). Mating and selection is allowed only within each subline for breeding purposes, while mating among sublines is only performed for the generation of a production population (PP) in order to limit the degree of inbreeding and presumably ID in deployment individuals. Sublining differs from multiple breeding population strategies in that the former is used only to manage inbreeding, while the latter usually deal with uncertainties of how to prioritize between several prospective breeding traits by applying different breeding objectives to different populations (Namkoong *et al.* 1988; Barnes 1995). The subline strategy has been used in many second or third generation tree improvement programs (Carson *et al.* 1990; McKeand and Bridgwater 1993; Baez and White 1997; Jayawickrama and Carson 2000; White and Carson 2004).

Another approach, called the nucleus breeding strategy, was originally proposed for livestock improvement programs with the aim of obtaining higher gains faster while maintaining long-term diversity and genetic gains. The breeding population of the nucleus breeding strategy is usually organized into two tiers: a small nucleus and a larger main tier. The nucleus tier is organized by selecting trees with the highest breeding values (∼10%) for intensive breeding, testing, and selection. The main tier contains the other candidates and a selection that were selected at lower intensities to avoid inbreeding and maintain genetic diversity, thus ensuring sustainable genetic gains in the long-term. Gamete transfer is allowed between these two tiers to bring gain and diversity. Several tree breeding programs have used this scheme (Cotterill and Cameron 1989; White 1993; McKeand and Bridgwater 1998; White *et al.* 1999).

A fundamentally different approach is the inbred and hybrid breeding strategies commonly used in crop breeding (Allard 1999). Since Shull (1909) and East (1909) first developed this idea in order to produce uniform and highly productive maize (*Zea mays* L.) hybrids, selfing and subsequent cross-breeding has been a main breeding method for the improvement of many agronomic species (Hallauer and Miranda 1981). Assuming that deleterious recessive alleles are the main cause underlying ID, it should be possible to eliminate these recessives and fix the favorable dominant alleles by applying systematic inbreeding in combination with directional selection, so called purging (Barrett and Charlesworth 1991; Hedrick 1994). Inbreeding depression would, thus, decline across generations as purging effectively removes deleterious alleles.

Selfing as a breeding tool for forest trees was first advocated five decades ago (Matthews and McLean 1957; Bingham 1973; Barker and Libby 1974). As tree breeding has progressed into second and third generations, the use of selfing and sib-mating as a breeding tool has been debated due to the growing interest in small elite breeding populations (Williams and Hamrick 1996; Williams and Savolainen 1996; Wu *et al.* 1998). Nonetheless, the long generation turnover and the observed severe ID have deterred tree breeders from using this approach.

Although the aforementioned breeding strategies, with their conceptual short- and long-term advantages and disadvantages, have been proposed for various tree breeding programs, no detailed comparative genetic studies have been carried out to quantify the short- (2–5 generations) and long-term (15–20 generations) genetic consequences of their implementation. Such comparative studies can only be done through simulation approaches, of which the generally used infinitesimal model does not allow the tracing of purged unfavorable alleles and cannot realistically emulate how recessive/dominant alleles would induce ID. Only locus-based models have the capability to track the behavior of individual alleles under a nonadditive situation and emulate more complex phenomena such as linkage disequilibrium (Hedrick 1994; Fu *et al.* 1998).

The objective of this study is to compare breeding strategies used for improving quantitative traits under additive and nonadditive modes of inheritance. For this, we set up a finite locus genomic model to simulate the various strategies and to examine relevant population parameters, the genetic gain in breeding and PPs, fixation of unfavorable or recessive alleles, and accumulation of inbreeding. We also assessed whether certain systematic inbreeding methods could be suitable to overcome inbreeding depression by purging of ID and unfavorable alleles given a certain range of conditions.

## Methods

### Breeding strategies studied

**Figure 1:**
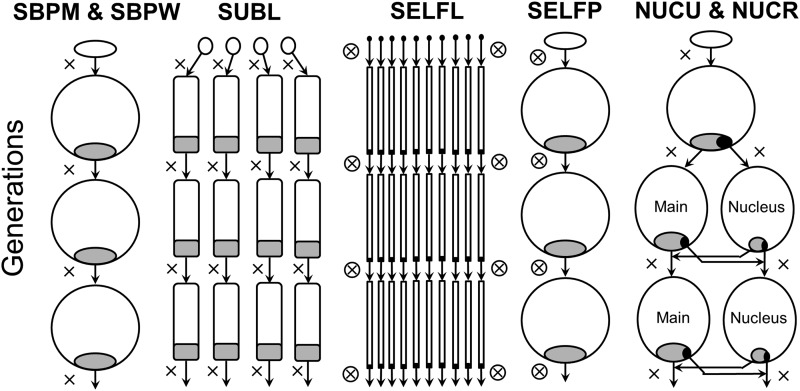
Schematic description of single population (SBPM and SBPW), sublines (SUBL), selfing in lines (SELFL), selfing in a single population (SELFP) and nucleus breeding strategies (NUCU & NUCR). Proportions selected for breeding are shaded in gray and black. Outcross and self matings are denoted with × and ⊗, respectively.

We compared seven breeding strategies ordered within four main categories (Figure 1): *i)* two single breeding population strategies (SBP), *ii)* one subline strategy (SUBL), *iii)* two nucleus strategies (NUC), and *iv)* two selfing and cross-breeding strategies (SELF). All strategies comprised a founder population of 192 unrelated individuals and in each generation a total of 9600 progenies were generated, of which 192 individuals again were phenotypically selected for further breeding. Single pair-mating with random allocation of parents was used for all strategies except for selfing strategies and for the main tier of nucleus strategies where open pollination was simulated (a random set of selected fathers were mated to each mother). Details of the different breeding strategy designs can be seen in Table 1.

**Table 1:** Detailed input parameters of the investigated breeding strategies

	Single BP (SBPM, SBPW, SELFP)	Sublines (SUBL)	Selfing in Lines (SELFL)	Unrestricted Nucleus Breeding (NUCU)		Restricted Nucleus Breeding (NUCR)	
Breeding population (BP) characteristics							
No BPs	1	4	192	Nucleus	Main	Nucleus	Main
No progeny							
Per BP	9600	2400	50	4800	4800	4560	5040
Selected for each BP	192	48	1	20	168	20	144
Selected for transfer	—	—	—	24	4	24	4
Selection intensity for BP	2%	2%	2%	0.42%	3.5%	0.44%	2.86%
Selection intensity for transfer	—	—	—	0.5%	0.08%	0.53%	0.08%
Elite selection to generate production population (PP)							
BPs selected	1	4	24	Nucleus		Nucleus	
No progeny selected/BP	24	6	1	24	0	24	0
Among-BP selection intensity	100%	100%	12.5%	—		—	
Within-BP selection intensity	0.25%	0.25%	2%	0.5%	—	0.53%	—

For the purpose of our study, we used the single breeding population and mass selection (SBPM) as a reference strategy. This was compared to an alternative single breeding population strategy where selection was performed only within-family (SBPW) which produces equal contributions among families and implicitly limits the mating of related individuals (*e.g.*, traditional Swedish tree breeding strategy, Danell *et al.* 1993). Comparisons were also made to a four sublines (SUBL) strategy where 48 individuals out of 2400 were selected within each subline, to selfing based on single seed descent within 192 lines of 50 progenies each (SELFL), and to selfing combined with mass selection in a single progeny population (SELFP). In addition, two nucleus strategies (NUCU and NUCR) were studied, which both included main and nucleus tiers, using different selection intensities and with the possibility to select parents for transfer from the main to the nucleus or vice versa. The two nucleus strategies differed in the sense that the very best individuals could be selected both for parentage and for transfer in the unrestricted nucleus strategy (NUCU), while in the restricted nucleus strategy (NUCR) unique individuals had to be chosen either for parentage or for transfer. The aim was to generate 4800 progenies for both the nucleus and main populations, but for NUCR this was not possible because the comparable setup would have required a noninteger number of progenies being generated per cross or per parent in either the nucleus or main tiers. Therefore, the number of progenies in the main tier had to be increased somewhat at the expense of the nucleus (see Table 1 for details).

For breeding populations (BP), an overall selection intensity of 2% at each generation was applied. However, to easily compare breeding strategies with respect to output performance we also simulated the generation of a PP by selecting and mating a total of 24 elite trees from BPs at each generation (overall selection intensity of 0.25%). Elite selections for the PP were evenly distributed among BPs, except for the nucleus strategies where selections were made only from the nucleus, and SELFL where only the best 24 inbred lines (*i.e.*, 24 BPs) were selected.

### Simulation theory and genomic setups

A locus-based simulation software (Metagene) using a finite loci model was previously developed at INRA (Sánchez *et al.* 2007; http://www.igv.fi.cnr.it/noveltree). The initial software was adapted to the study of breeding strategies dealing with adverse genetic correlations (Hallingbäck *et al.* 2014) but was further expanded to simulate the short and long-term behavior of populations subjected to the breeding strategies of this study (Supporting Information, File S1, File S2, File S3, File S4, File S5, File S6, File S7, File S8, and File S9).

The virtual tree genomic framework was designed to comprise 100 biallelic loci (alleles *B* and *b*) affecting a trait of our interest. ID of varying severities was simulated by regulating the degree of dominant allele action at each of the 100 loci. In principle, three different scenarios were investigated which comprised: *i)* allelic effects of a purely additive nature (*a* = 1, *d* = 0) thus emulating the complete absence of ID, *ii)* partially dominant allele effects (*a* = 1, *d* = 0.5*a*) leading to a relatively mild ID, and *iii)* completely dominant allele effects (*a* = 1, *d* = *a*) implying severe ID. All loci were set to be physically unlinked (independent assortment of alleles across loci) and no epistatic interactions were simulated. In addition to the 100 loci controlling the trait, an equal number of multi-allelic neutral loci were incorporated in the framework. For those loci, each founder was given a unique set of alleles thereby enabling the calculation of probabilities by descent and thus the inbreeding level (*F*) of individuals and populations. Virtual individuals and breeding populations (genotypes and genotypic values) were created with respect to the designed genomic framework and breeding strategies. Phenotype values were created by adding randomly sampled environmental deviates to the genotypic values. Environmental deviates were sampled from a normal distribution with zero mean and variance calibrated to produce an initially narrow sense heritability (*h^2^*) of 0.3 for the total founder population. Genetic gain accumulated for a given population at generation *t* was calculated as the increase of average population genotypic value at generation *t* compared to the mean value of the founders.

The degree of ID is a dynamic property dependent on allele and genotypic frequencies as well as the degree of dominant allele action. Consequently, in order to continuously monitor the capacity of a population to express ID at any given time and situation, we calculated µ*_D_*, which is the average genetic gain due solely to dominance deviations only expressed at loci where the individuals were heterozygous:

$$\mu_{D}=\frac{1}{N}\overset{N}{\underset{i=1}{\sum }}\overset{n}{\underset{j=1}{\sum }}d_{j}H_{ij}$$ (Equation 1)

where *N* is the number of individuals in the population, *n* is the number of loci, *d_j_* is dominant gene action at locus *j*, and *H_ij_* is a binary indicator of whether individual *i* is heterozygous (*H* = 1) at locus *j* or not (*H* = 0). Assuming the absence of epistasis, linkage disequilibrium (LD), and any directed selection the theoretically expected ID capacity (Falconer and Mackay 1996) is:

$$E(\mu_{D})=2\overset{n}{\underset{i=1}{\sum }}\left(1-F_{i}\right)p_{i}(1-p_{i})d_{i}$$ (Equation 2)

where *F_i_* and *p_i_* is the locus-wise inbreeding coefficient and favorable allele (*B*) frequency for locus *I*, respectively. Initial settings for the different genomic scenarios are summarized in Table 2.

**Table 2:** Schematic view of the simulated genomic scenarios, their allele effects (*a* and *d*), their expected initial ID capacity, E(µ*_D_*) as calculated by Equation 2 and in relation to the additive genetic standard deviation, E(µ*_D_*)/σ*_A_*

	Dominance Level Scenarios		
	Complete Additivity	Partial Dominance	Complete Dominance
Intermediate allele frequencies scenario			
Effects, all 100 loci	*a* = 1, *d* = 0	*a* = 1, *d* = 0.5	*a* = 1, *d* = 1
E(µ*_D_*)	0.0	25.0	50.0
E(µ*_D_*)/σ*_A_*	0.0	3.5	7.1
U-shaped allele frequencies scenario (*N_e_* = 192)			
Effects, all 100 loci	*a* = 1, *d* = 0	*a* = 1, *d* = 0.5	*a* = 1, *d* = 1
E(µ*_D_*)	0.0	13.9	25.1
E(µ*_D_*)/σ*_A_*	0.0	2.0	3.6
Major & minor loci scenario			
Effects, 20 major loci	*a* = 5, *d* = 0	*a* = 5, *d* = 2.5	*a* = 5, *d* = 5
Effects, 80 minor loci	*a* = 1, *d* = 0	*a* = 1, *d* = 0	*a* = 1, *d* = 0
E(µ*_D_*)	0.0	10.4	20.8
E(µ*_D_*)/σ*_A_*	0.0	1.5	2.9

Notably, the ID capacity is theoretically equivalent to the linear regression coefficient of average phenotype on *F*, which is frequently estimated and reported in empirical inbreeding studies (Falconer and Mackay 1996). By searching the literature, we observed that empirical estimates are usually given in percentages related to the mean of the noninbred population (100 × µ*_D_*/µ*_F_*_= 0_). Consequently, these estimates are easily divisible with the percentage additive genetic coefficient of variation (*CV_A_* = 100 × σ*_A_*/µ*_F_*_= 0_) producing an estimate of the ID capacity per unit additive genetic standard deviation (µ*_D_*/σ*_A_*) fully comparable to corresponding values for the genomic scenarios of this study (see E(µ*_D_*)/σ*_A_* in Table 2). *CV_A_* estimates from the meta-analysis of Cornelius (1994) were used for the study (*e.g.*, 10% for height and diameter growth and 20% volume). Thus, the initial ID capacities used in our simulations could be compared to empirical observations made for forest trees and the realism of the ID severity for the simulated genomic scenarios could be assessed.

The standard setting for the virtual genomic framework comprised all 100 loci exerting equally small additive effects (*a* = 1) on the trait of interest and that the founder population would have intermediate allele frequencies (E(*p_i_*) = 0.5). However, to test the robustness of the results with respect to allele frequencies, an alternative setup was designed where initial allele frequencies were set to follow a neutral model (U-shaped) distribution adjusted to an effective population size equal to the number of founders (*N_e_* = 192, see Hill *et al.* 2008). Additional setups were also created where 20 loci were set to have much larger effects (*a* = 5, major loci) than the other 80 loci (*a* = 1, minor loci). In this scenario, dominance effects were only assigned to the 20 major loci emulating a situation where the effects of ID origins only form a limited but influential part of the genome.

One should note that variation in parameters such as allele effects and frequencies, set by choice or as a result of random sampling, will affect the initial additive genetic variance (σ*_A,init_^2^*), which is paramount for the success of phenotypic selection. Therefore, in order to be able to compare breeding strategies and scenarios at equal initial conditions, all resultant parameters were scaled to correspond to an initial σ*_A_^2^* at 50 for the founder generation (variance type parameters where multiplied by 50/σ*_A,init_^2^* and mean type parameters by √50/σ*_A,init_*). Simulations were run 500 times and averages of these are reported.

### Data availability

No experimental raw data were used or generated. The simulation software (File S1, File S2, File S3, File S4, File S5, File S6, File S7, File S8, and File S9) is deposited at http://www.upsc.se/resources/databases-a-software.html and can be downloaded from file folder “genomic simulation” for execution.

## Results

### Increasing inbreeding coefficient in breeding populations

**Figure 2:**
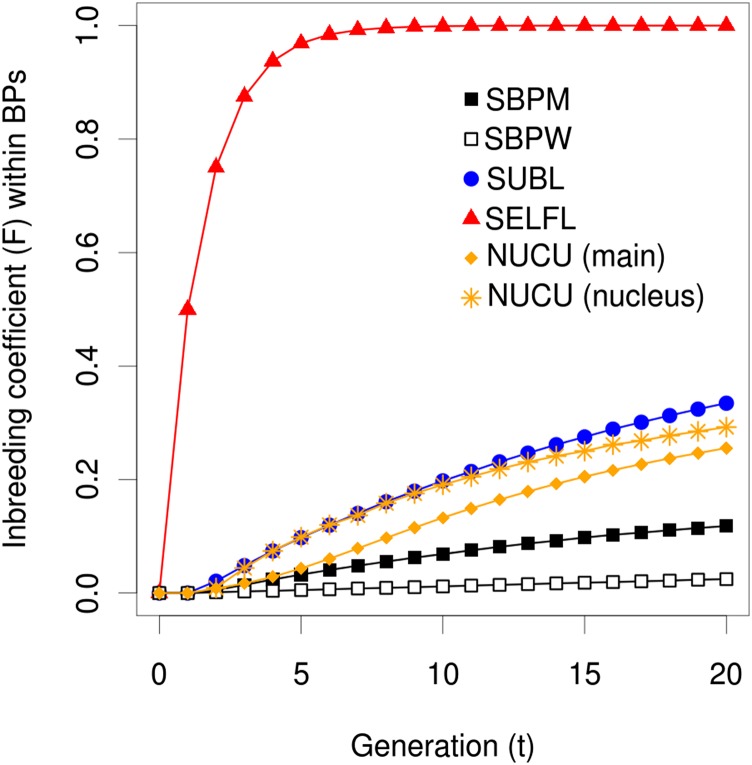
Average inbreeding coefficients over generations within breeding populations for additive allele action scenario. The inbreeding development of SELFP was very similar to that of SELFL and is thus not shown. NUCU, nucleus breeding strategies; SBPM and SBPW, single population; SELFL, selfing in lines; SELFP, selfing in a single progeny population.

The inbreeding coefficient (*F*) of the BPs was plotted for the seven breeding strategies (Figure 2). Figure 2 shows only the results of the additive allele action scenario and intermediate allele frequencies because the inbreeding development under the other scenarios was very similar. Both selfing strategies (SELFL and SELFP) showed the extreme and expected asymptotic convergence toward complete inbreeding and were completely similar to each other. In contrast, the inbreeding avoidant SBPW exhibited hardly any increase in *F* at all. SBPM also accumulated lower levels of inbreeding relative to nucleus and subline breeding populations. Although the nucleus tiers of the nucleus breeding strategies (NUCU and NUCR) accumulated inbreeding quicker than the corresponding main tiers, the differences in *F* were not very large and decreased by time. This indicates that nucleus strategy transfers are effective in keeping inbreeding low and maintaining genetic diversity in the nucleus population. The inbreeding of the unrestricted nucleus strategy (NUCU) increased slightly quicker than that of the restricted nucleus strategy (NUCR, not shown), but with respect to all other parameters NUCU behaved almost identically to NUCR. Hence, only NUCU will be shown and discussed further in this study.

### Genetic advance in breeding populations

**Figure 3:**
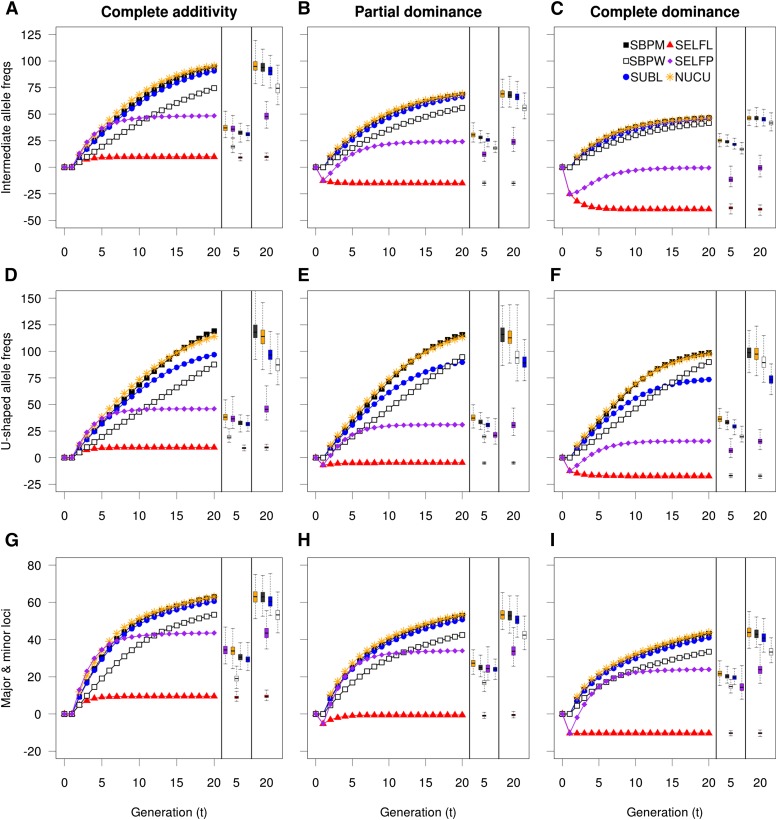
Average genetic gain over generations in breeding populations under different conditions with respect to level of dominance (among columns) or to allele frequencies and effect sizes (among rows). Variation in average gain over simulations (500) is shown with boxplots (one per strategy) in separate fields at the right side in each subplot, for generations 5 and 20. Only the nucleus tier of NUCU is shown. NUCU, nucleus breeding strategies; SBPM and SBPW, single population; SELFL, selfing in lines; SELFP, selfing in a single progeny population; SUBL, sublines.

Genetic gains in breeding populations were more complex than the inbreeding coefficient accumulation (Figure 3). In the scenario of complete additivity, the selfing strategy performed in a single large population (SELFP) exhibited the highest genetic gains during the first 4four to five generations, after which no further substantial gains were made. In contrast, the single seed descent selfing strategy (SELFL) showed the worst performance in all generations. When dominance was present, both selfing strategies experienced ID losses during the early generations, apparently creating permanent gaps in gain relative to the other strategies. Nucleus (NUCU), subline (SUBL), and single breeding population (SBPM) strategies all performed better in the long-term, with NUCU and SBPM having a tendency to perform slightly better than SUBL. Under major loci scenarios (Figure 3, G–I), the selfing strategies performed relatively better, notably SELFP. The results for U-shaped allele frequency scenarios (Figure 3, D–F) were fairly similar to those of intermediate allele frequencies apart from ID effects being milder as expected from E(µ*_D_*) (Table 2). For the U-shaped allele frequency scenarios, the gap in genetic gain between SUBL and the two better strategies (SBPM and NUCU) was larger than under intermediate allele scenarios, and in the very long-term there was even a tendency for SUBL to perform worse than within-family selection (SBPW). In conclusion, SBPM and NUC were the best all round strategies in terms of gain rendering, while SELFL always produced the least gains.

### ID capacity and the fate of detrimental alleles

**Figure 4:**
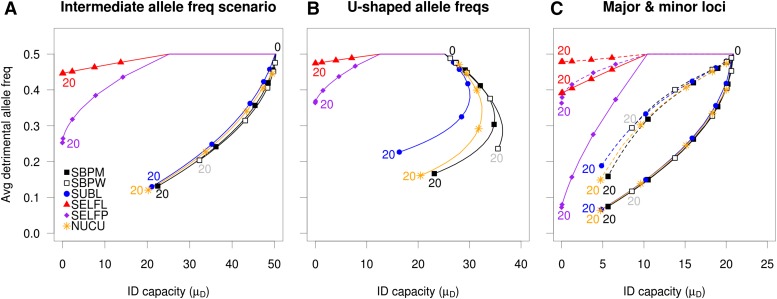
Development of average detrimental allele frequency and the inbreeding capacity (*µ_D_*) in the breeding population under conditions of complete dominance combined with intermediate allele frequencies (A), U-shaped allele frequencies (B) and major and minor effect loci (C). Symbols are shown for generations 2, 3, 5, 10, and 20 (marked), the nucleus population of NUCU is shown and minor loci are depicted with dashed lines. NUCU, nucleus breeding strategies; SBPM and SBPW, single population; SELFL, selfing in lines; SELFP, selfing in a single progeny population; SUBL, sublines.

From a breeding context, successful purging of the genetic load should encompass the decrease of unfavorable allele frequencies, as well as the ID capacity itself (µ*_D_*). From Figure 4 it was evident that the selfing strategies (SELFL and SELFP) showed the fastest and most complete purge of ID capacity while the inbreeding avoidant breeding strategy (SBPW) exhibited the slowest decline, thus conserving the ID capacity in the long-term. However, the selfing strategies, especially SELFL, were largely unsuccessful in purging the detrimental alleles because their average frequencies became largely fixed at considerable levels after five generations of inbreeding. In contrast, the corresponding detrimental allele frequencies of the other strategies never stabilized, but decreased continuously throughout all 20 simulated generations. Excepting the major loci scenario, the SBPM, SUBL, NUCU, and NUCR strategies were even able to decrease the detrimental allele frequencies to a greater extent than SELFL and SELFP, while still showing a µ*_D_* lower at generation 20 than at the outset. This behavior is consistent with so-called slow purging, which can be performed without the systematic mating of related individuals. Only scenarios with complete dominance are shown here because the partial dominance scenario results were similar in all respects, excepting only the scale of the ID capacity. Additive scenarios, as expected, never exhibited any ID.

In contrast to the monotonic µ*_D_* decrease for all strategies under intermediate allele frequency scenarios (Figure 4, A and C), initial increases in µ*_D_* could be observed for several strategies under the U-shaped allele frequency scenario (Figure 4B). These increases reversed signs after reaching maxima that occurred at different levels and generations among strategies. This can be explained by the fact that, given intermediate allele frequencies, the ID capacity will always be at its theoretical maximum (Equation 2), while for U-shaped allele frequencies the loci with low allele frequencies may be brought closer to intermediate frequencies as a result of selection, thus increasing µ*_D_*. Under the major–minor loci scenario (Figure 4C), SELFP was actually able to perform a fast purge of the major unfavorable alleles, matching that of the nonselfing strategies. However, the SELFP purging of the corresponding minor alleles was much less successful in comparison to that of the outcrossing strategies.

**Figure 5:**
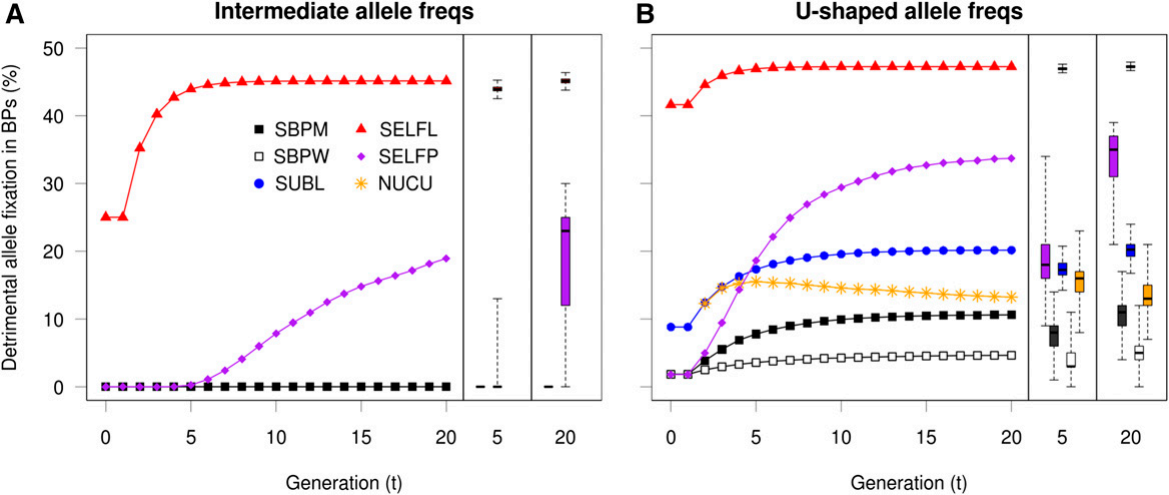
Percentage of detrimental allele fixation (out of 100 loci) under conditions of complete additivity combined with intermediate allele frequencies (A) and U-shaped allele frequencies (B). Under intermediate allele frequencies, all strategies except SELFL and SELFP exhibited virtually no allele loss and thus only SBPM is shown among them. Variation in percentage of detrimental fixation over simulations (500) is shown with boxplots in separate fields at the right side in each subplot, for generations 5 and 20. The nucleus population of NUCU is shown. NUCU, nucleus breeding strategies; SBPM and SBPW, single population; SELFL, selfing in lines; SELFP, selfing in a single progeny population; SUBL, sublines.

As previously mentioned, the selfing strategies quickly decreased their ID capacity but also successively lost the ability to continuously decrease the unfavorable allele frequency due to considerable fixation (Figure 5). Indeed, at intermediate frequencies, SELFL showed high levels of unfavorable fixation at the outset and this fixation increased to 45% at generation 20 (Table 3). SELFP showed considerable fixation from generation 5 onwards, while no fixation was observed for other (nonselfing) breeding strategies even after 20 generations (Figure 5). The high initial fixation for SELFL was expected, as each selfing line (BP) was generated by one single individual sampled from a founder pool at approximate Hardy-Weinberg equilibrium with intermediate allele frequencies (*P* = 0.5). Thus the expected probability of unfavorable fixation at the outset was (1-*p*)^2^ = 0.25. For major loci, fixation in SELFL at generation 20 was somewhat lower (39–40%), likely due to their greater individual contribution to the additive genetic variance, making them easier targets for selection. The minor loci counterparts, however, showed a comparatively greater degree of fixation as their small effects were overshadowed by that of the major loci, and their fate was thus mainly dominated by drift (SELFL and SELFP in Figure 4). The fixation of unfavorable alleles was affected very little by the level of dominance used (Table 3) and, therefore, only the completely additive scenario is shown in Figure 5.

**Table 3:** Average number of alleles unfavorably fixed at generation 20 for the SELFL strategy under different scenarios

	Additive (*d* = 0)	Part Dominance (*d* = 0.5*a*)	Complete Dominance (*d* = *a*)
Allele frequency = 0.5	45.1	45.0	44.6
U-shaped frequencies	47.3	47.3	47.4
Major loci (*a* = 5)*^a^*	40.3	39.3	39.1
Minor loci (*a* = 1)*^a^*	47.9	47.9	47.8
Intensive selection*^b^*	44.1	43.9	43.5

a Allele fixations for major and minor loci are derived from the same set of simulations.

b Intermediate allele frequencies scenario with fourfold intensified selection.

Under U-shaped allele frequencies, all breeding strategies experienced unfavorable allele fixation (Figure 5B) likely due to these alleles having frequencies close to fixation from the start. Another possibility is that unfavorable alleles at any frequency could be hitchhiked by rapidly expanding favorable alleles in the population. Initial fixation for SUBL and the nucleus tiers of the two nucleus strategies were higher than for the SELFP, although SELFP overtook SUBL and NUCU after only five generations. The NUC strategies were unique in being able to decrease fixation in their nucleus tiers by time. Such fixation decreases were obviously facilitated by the receipt of fresh supplies of alleles from the main tier. When allele frequencies were U-shaped, the SBPW strategy appeared best suited to keep detrimental alleles from fixation.

Early severe inbreeding with lesser impact on the adult stage would be easier to deal with and could be used to purge inbreeding depression at seed and seedling stage, potentially applying selection of great intensity (by increasing the population size). Therefore, an additional SELFL strategy was designed where the selection was intensified four times in comparison with the normal SELFL strategy. Unfavorable allele fixation for such a strategy was nonetheless very similar to the normal SELFL strategy with respect to the unfavorable allele fixation (44%, Table 3).

In summary, selfing strategies appeared to substantially reduce their capacity to show ID, but this happened partly at the expense of fixing high percentages of unfavorable variants. At the opposite extreme, SBPW kept high levels of potentially harming alleles hidden at the heterozygote state, with little unfavorable fixation. NUCU and SBPM appeared as the best compromises among tested strategies due to their slow but continuous purging of both ID capacity and unfavorable alleles, and by avoiding the fixation of unfavorable alleles.

### Evolution of genetic variance in breeding populations

The additive genetic variance declined with the advancement of generations of selection (Figure S1). Selfing strategies showed the fastest decline while the within-family selection strategy declined the least. The theoretical expectation of genetic variance distributed between lines (2*F*σ*_0_*^2^) and within lines ((1-*F*)σ*_0_*^2^) was observed in this study where, at generation 1, the within-line selection strategy of SELFL could access only 50% of the additive genetic variance available to the nonselfing strategies, whereas the selfing within a single population strategy could access an additive genetic variance 50% larger than that of the nonselfing strategies as a consequence of the selfing itself. The SELFP peak in σ*_A_*^2^ at generation 1 most probably also contributed to the higher genetic gains observed for SELFP (Figure 3) in the early generations, since genetic gain is a function of additive genetic variance (Crow and Kimura 1970).

### Inbreeding coefficient trend in the production population (PP)

The change of the inbreeding coefficient in the PP was similar to that of the breeding populations for SELFP, but opposite to that for SELFL (Figure S2). The selfing strategy performed in a single population (SELFP) was completely unable to keep inbreeding low in the PP, as the best individuals were chosen without restrictions from one single population in which inbreeding increased very quickly (Figure 2). On the other hand, inbreeding levels in the PP for the SELFL strategy was among the lowest due to the selection of unrelated individuals for hybridization.

### Genetic gain in the PP

**Figure 6:**
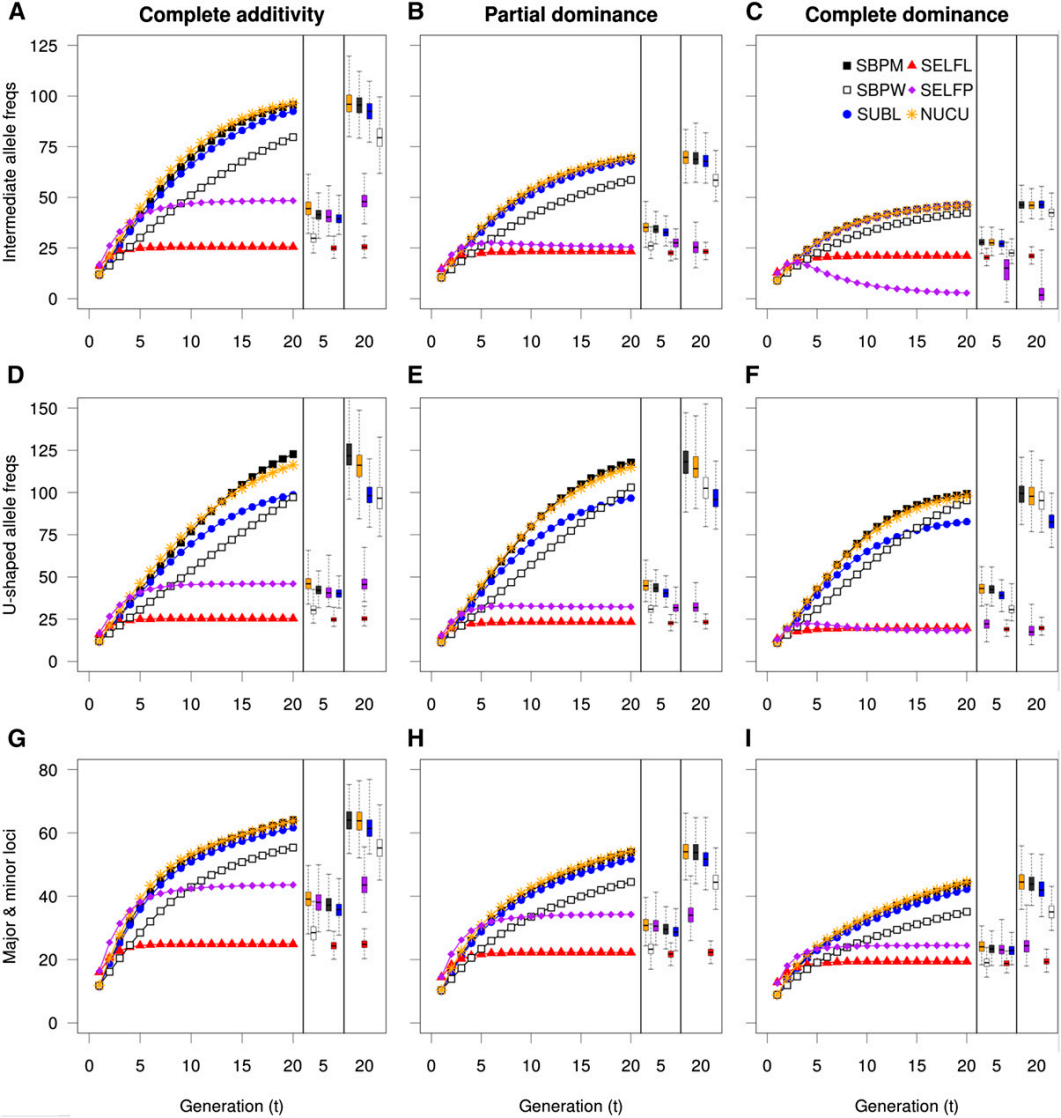
Genetic gain in the production population. Genetic gain in the production population under different conditions with respect to allele level of dominance (columns) and to allele frequencies and effect sizes (rows). Variation in genetic gain is shown with boxplots in separate fields at the right side in each subplot, for generations 5 and 20. Among the nucleus strategies only NUCU is shown. NUCU, nucleus breeding strategies; SBPM and SBPW, single population; SELFL, selfing in lines; SELFP, selfing in a single progeny population; SUBL, sublines.

Genetic gain in the PP is paramount as it describes the final gain output available to commercial forestry. Genetic gains in the PP (Figure 6) were somewhat different from those of the breeding populations. First of all, the selfing strategies (SELFL and SELFP) exhibited the highest genetic gain in the first few generations and the gains were higher for SELFP than for SELFL. However, this superiority of SELFP and SELFL was weaker and shorter lived at increased dominance levels or at U-shaped allele frequencies. When simulating 20 major loci, the selfing strategies improved a little in comparison to the other strategies. After generation 5 for SELFL and generation 10 for SELFP, genetic gains for the selfing strategies were considerably lower than for other breeding strategies regardless of the genomic setup.

The genetic gain of SELFL was clearly hampered by the ineffective within-line selection and by the unfavorable allele fixations experienced at the breeding stage. This pattern was evident in spite of PP genetic gain being given an extra boost produced by heterosis and by the selection of the 24 best lines. The selection in the SELFP BP was much more efficient with respect to gains (Figure 3), but because SELFP lacked any population subdivision that would limit the coselection of related individuals (self fullsibs) the possibility to acquire heterosis was gradually lost. That is likely the reason why, in scenarios of severe ID, SELFP actually performed worse than SELFL with respect to long-term genetic gain in the production population (Figure 6C).

Under all scenarios, the nucleus (NUC) and single breeding population strategy with mass selection (SBPM) accumulated the highest long-term genetic gains and, given the replicate variation, they also performed equally well. SUBL and SBPW were consistently inferior in comparison to the NUC and SBPM strategies, both in the short and long-term perspective despite their lower PP inbreeding coefficients (Figure S2). However, the inferiority of SUBL was very slight under intermediate allele frequencies (Figure 6, A–C) and major loci scenarios (Figure 6, G–I), and SBPW showed a relatively better long-term performance under the scenario where complete dominance was combined with U-shaped allele frequencies. In conclusion, NUC and SBPM were the best overall performing strategies, while SELFP could have some advantage at short term and whenever dominance was not important. The worst overall performer in delivering gain at the PP was SELFL.

### Genotypic variance in the PP

**Figure 7:**
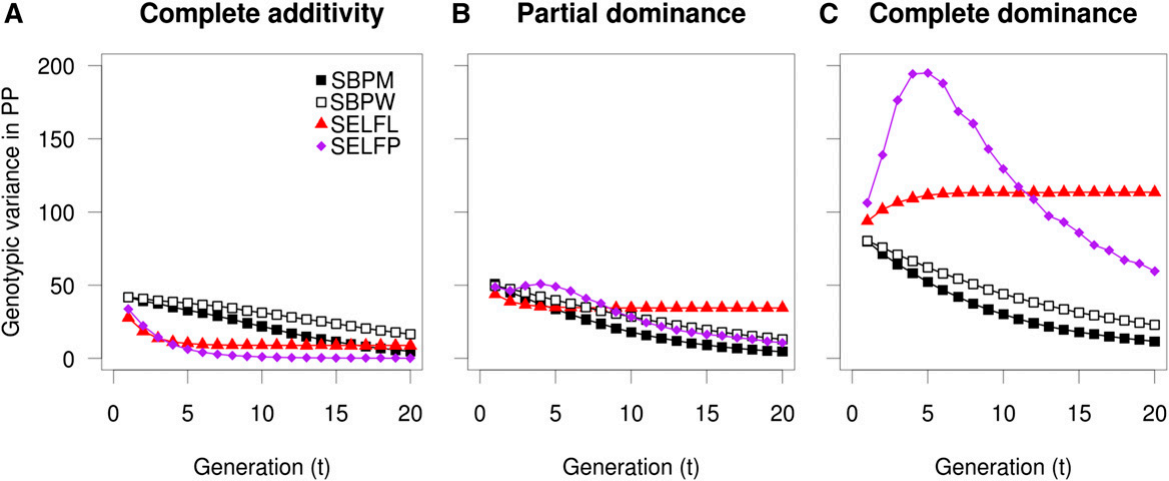
Development of genotypic variation (σ*_G_^2^* = σ*_A_^2^* + σ*_D_^2^*) in the production population. Development of genotypic variation (σ*_G_^2^* = σ*_A_^2^* + σ*_D_^2^*) in the production population under conditions of intermediate allele frequencies combined with complete additive gene action (A), partial dominance (B) and complete dominance (C). The developments for subline (SUBL) and nucleus (NUC) strategies were extremely similar to that of SBPM and are thus not shown. SBPM and SBPW, single population; SELFL, selfing in lines; SELFP, selfing in a single progeny population.

The genotypic variation in the PP is an important measure of the uniformity of the deployed genotype mix. In the completely additive scenario, the genotypic variance decreased by time for all strategies and the decline was fastest for the selfing strategies (Figure 7A). However, when any degree of dominance effect was included, the genotypic variance in the selfing strategies decreased at a slower rate than under the additivity (Figure 7, B and C). The genotypic variance even increased, in particular for SELFP, during the first generations (Figure 7, B and C). These increases in genotypic variances for SELFL and SELFP could be explained by linkage disequilibrium (LD) generated through the admixture of random mating. Under complete dominance, outcrossing of parent lines would generate progeny with considerable heterosis gains (due to different loci being favorably/unfavorably fixated in different lines), while selfing would just reproduce the depressed genotype of the elite parent line. By separating the genetic LD-covariances from genic variances, the increases in σ*_G_^2^* were indeed observed to be caused by LD (not shown).

## Discussion

Scientific and theoretical examination of breeding strategies for tree species is more relevant than for other crops due to the impracticality of performing multiple generation experiments on long-lived tree species. Furthermore, most tree breeding programs are still at a comparatively early stage among domesticated species. Therefore, designing optimal breeding methods is essential for ensuring short- and long-term genetic gain. Traditionally, the design of breeding strategies was regarded as half science and half art (Shelbourne *et al.* 1986) due to the complex admixture of theoretical principles, operational knowledge, and the accounting of biological constraints. This complexity is one of the reasons why the breeding strategy for radiata pine, which is far advanced in terms of breeding, evolved with gained experience from a nucleus breeding strategy to a subline strategy, and then to a single population with rolling-front operation (Cotterill 1989; Borralho and Dutkowski 1998; White and Carson 2004; Wu *et al.* 2007).

How to deal with inbreeding effectively is a central issue in the decision of which strategy is best for long-term tree breeding. Inbreeding and diversity can be managed implicitly through design of the breeding scheme, or explicitly when selection and mating decisions are to be taken. This paper deals with the former type of management being the first important step when designing a breeding strategy. Explicit management can be added later on to the design for further efficiency. In order to efficiently manage advanced breeding programs, several strategies were proposed or adopted from animal and crop breeding. Among these strategies, two general approaches are noteworthy: *i)* inbreeding should be actively avoided and the genetic load could be slowly purged by gradual increase of favorable allele frequencies as a result of selection; and *ii)* fast purging could be performed using deliberate inbreeding as a tool in combination with directed selection.

### Comparing simulated ID with that observed for conifer species

Inbreeding depression has been observed in forest trees for number of sound seeds, seedling performance, adult growth, and fecundity. The ID for survival of seedlings and adults was often observed to be considerable (43–93%) (Williams and Savolainen 1996) and the corresponding depression of adult fecundity ranged from a small loss of 6.7% in *Pinus radiata* (Wu *et al.* 2004b), to high (53%) in *Pinus pinaster* after selfing (Durel *et al.* 1996). The ID for the adult height growth after selfing also varied a lot among species and ranged from low (9%) in *P. radiata* (Wilcox 1983) to high (61%) in *Picea abies* (Eriksson *et al.* 1973). In the context of this study, ID observations of field growth are particularly interesting because estimates of the ID capacity per additive genetic standard deviation (E(µ*_D_*)/σ*_A_*) are comparable to the corresponding parameters used in our simulations (Table 2). From a literature survey focusing on growth traits (Table 4), species such as *P. abies*, *Picea glauca* and *Pseudotsuga menziesii* were found to exhibit severe ID (µ*_D_*/σ*_A_* in the range 4.6–8.8) fairly close to the initial ID capacity of the complete dominance with intermediate allele frequencies scenario (7.1) used in this study. At the other end, the milder ID observed for *P. radiata* and *Pinus banksiana* (1.7–3.2) were more similar to those of the partial dominance with U-shaped allele frequencies (2.0) or the major loci complete dominance (2.9) scenarios. In conclusion, the genomic scenarios designed and simulated in this study exhibited an initial capacity to show ID that compared well with the ID observed in a collection of forest tree species.

**Table 4:** Estimates of inbreeding depression in relation to the additive genetic standard deviation (µ*_D_*/σ*_A_*) for growth traits calculated from literature assuming additive coefficients of variation (*CV_A_*) of 10% for tree height and diameter and 20% for volume (Cornelius 1994)

Species	Tree Height	Diameter	Volume	Reference
*Picea abies*	4.6–6.8	7.4–7.6	—	Skrøppa 1996
*Picea glauca*	6.0	—	—	Doerksen *et al.* 2014
*Pseudotsuga menziesii*	4.8–5.8	8.8	7.2	Sorensen 1999
*Pinus elliottii*	2.4–5.2	3.7–9.4	3.6–8.2	Matheson *et al.* 1995
*Pinus sylvestris*	3.6–6.2	—	3.5–5.8	Lundkvist *et al.* 1987
*Pinus pinaster*	3.5	5.1	4.4	Durel *et al.* 1996
*Pinus taeda*	3.5–4.2	—	3.2–3.3	Ford *et al.* 2015
*Pinus banksiana*	2.4–3.2	—	—	Rudolph 1981
*Pinus radiata*	1.7	2.3–2.8	3.0	Wilcox 1983
*Pinus radiata*	—	2.7	—	Wu *et al.* 1998

Ranges are given for traits assessed at several sites or timepoints.

### Performance of the nonselfing strategies

In this study, we observed that the nucleus breeding and single breeding population with mass selection strategies were the best in terms of long-term genetic gain. The superiority of SBPM and NUC was observed in both breeding and PPs (Figure 3 and Figure 6), regardless of the mode of allele effects and despite exhibiting inbreeding coefficients in the PP higher than those of other nonselfing strategies (Figure S1). The reasons for the successful performance of NUC could be the combination of intensive selection and continuous supplies of genetic diversity from the main to the nucleus tier. Also, NUC appeared to purge both detrimental alleles and ID capacity at a slightly higher rate than SBPM (Figure 4) making it less susceptible to ID in the long-term. Although the NUC strategy exhibited higher levels of unfavorable fixation than SBPM under the U-shaped allele frequency scenario (Figure 5), it was still able to eventually decrease this fixation due to the introduction of alleles preserved in its main tier. Consequently, for the purpose of genetic improvement and handling ID, nucleus breeding could be a strategy of choice, taking into account that it requires a smaller number of crosses to be performed per generation than SBPM. On the other hand, the different management of the main and nucleus populations and the logistics of genetic transfers between them may increase the operational burden in comparison to the more simplistic SBPM strategy.

While the PP genetic gains of the subline strategy were only slightly lower than those of NUC and SBPM under intermediate allele frequency and major loci scenarios, it performed considerably worse under the U-shaped allele frequency scenario due to higher degrees of unfavorable allele fixation in the relatively small sublines (Figure 5B). SUBL also showed faster inbreeding accumulation within sublines than that of NUC, SBPM, and SBPW. These observations, in combination with selection within sublines being unable to access the whole genetic variation of the population, might have contributed to the lower genetic gains observed. Our simulations consequently indicate that the subline breeding strategy is not the most suitable in terms of short- and long-term genetic gain in tree breeding.

The within-family selection strategy in a single breeding population was advocated for tree species with severe ID and high genetic loads (*e.g.*, *P. abies*, *P. menziesii*, *and Pinus sylvestris*, Table 4) because it maintains very low levels of inbreeding and avoids ID (Danell *et al.* 1993). Our simulations confirmed these characteristics (Figure 1 and Figure 4) and also indicated SBPW to be efficient in terms of keeping unfavorable alleles from fixation and to conserve the greatest amount of genetic variance. However, in most cases, SBPW also produced lower genetic gains and conserved higher frequencies of unfavorable alleles than other nonselfing strategies. Only for scenarios with increased risks of unfavorable allele fixation (U-shaped allele frequencies) did SPBW show a limited degree of superiority by producing greater gains than SUBL in the very long-term (15th generation and later). In conclusion, SBPW is better suited for genetic conservation purposes where great genetic diversity and variation are *per se* regarded as primary objectives and the genetic improvement is merely a secondary goal.

### Performance of the selfing strategies

Despite the generally observed ID of growth and fitness at the population level in conifers, inbreeding or selfing has nonetheless long been advocated as a breeding method due to its complete assortative mating, maximum efficacy of selection among lines, and as a means of increase uniformity within lines (Lindgren 1975; Wu *et al.* 2004a). However, from our current simulations using 100 loci with many minor effects, we observed that selfing strategies were always inferior in genetic gain in the long-term for both breeding and PPs. The rapid increase in *F* in the breeding lines was, however, not the main issue *per se*, as the ID losses were completely recovered in the PP by outcrossing and heterosis. Although the ID capacity quickly decreased following the first cycles of selfing (SELFL and SELFP), unfavorable alleles were still not effectively purged (Figure 4), nor were the genetic gains improved in the long-term.

The major issue for the selfing strategies was that the rapid fixation of favorable alleles was incidentally accompanied by fixation of unfavorable alleles that escaped directional selection (Figure 4 and Figure 5), a process that occurred even under scenarios of no ID (effects completely additive) and despite experimentation with increased selection intensities (Table 3). However, the selfing strategies were more successful in protecting a set of fewer major effect loci from unfavorable fixation. This is consistent with a simulation study (Wu *et al.* 2004a) where selfing strategies performed purging successfully under a scenario with few loci of major effects controlling the trait and lethality, preventing the fixation of unfavorable alleles. All these observations support the explanation that a selection pressure distributed among many loci, each of small effect, is unable to prevent unfavorable fixation due to considerable genetic drift. An alternative reason, however, is the fact that inbreeding occurred relatively fast, with little chance given to segregation to render new recombination of genotypes, thus limiting the opportunities for selection to tell unfavorable and favorable allele carriers apart.

Interestingly, the SELFP strategy demonstrated a greater potential for purging by applying selection both among and within lines. Indeed, some highly promising hybrids were produced in the PP population of the SELFP strategy during the first few generations. However, in the long-term, fixation of recessive alleles constitutes the greatest challenge for the SELFP strategy.

In conclusion, the fixation of unfavorable alleles rendered the SELFL and SELFP strategies largely unable to recover the early genetic losses incurred by ID. For species with severe ID such as *P. menziesii* (Sorensen 1999) and *Pinus elliottii* (Snyder 1972), the application of inbreeding and selfing breeding strategies would likely result in considerable fixation of recessive alleles. Given a severe ID, another potential risk is the generation of highly heterogenous planting material (the PP) due to LD-based genetic variation (Figure 7) unless the parental elite lines could be systematically prevented from selfing (Hallingbäck *et al.* 2014). Tree species or traits with a reduced number of effective loci or exhibiting milder ID such as *P. radiata*, *Pinus resinosa*, *Picea omorika*, or *Thuja plicata* (Fowler 1965; Koski 1973; Wilcox 1983; Wu *et al.* 1998; Russell and Ferguson 2008) might be more amenable to a selfing and cross-breeding approach. However, given the results of this study, considerable care in performing efficient selection or the use of large segregation populations also appears to be required in species with mild ID. The fixation of unfavorable alleles and rapid depletion of genetic variance and genetic gain from SELFL and SELP strategies also raises the question of whether the traditional inbreeding and cross-breeding methods used extensively in outcrossing crop species such as maize are optimal for long-term breeding.

### Prospects for future research

Apart from the comparisons between selfing and nonselfing strategies already mentioned, it was also observed that strategies featuring isolated breeding compartments (*e.g.*, SUBL and SELFL) performed relatively worse than strategies devoid of such structures (SBPM and SELFP) or permitted a certain gene flow across compartments (*e.g.*, NUC). It appears that the existence of isolated breeding compartments limits the choices when optimizing selection and mating decisions. Strategies devoid of compartment structures appear better suited for the implementation of optimal procedures that explicitly control inbreeding. For instance, selection in SBPM, NUC, or even SELFP could be enhanced by applying optimum contribution selection and minimum coancestry mating designs, which could optimize the balance between accumulated inbreeding and genetic gains (*e.g.*, Stoehr *et al.* 2008; Hallander and Waldmann 2009). Such improvements offer prospects for still better performance in terms of inbreeding control or purging than those investigated in this study.

Another interesting aspect that could affect the ultimate worth of inbred lines is the frequency and distribution of useful alleles in the progenitors. Initial allele frequencies from intermediate to high (*P* = 0.8) for the favorable alleles could make the situation for selfing more favorable than in our scenarios, as unfavorable fixation is then less likely. Hence, the selection of a superior base population could increase the probability of generating high quality inbred lines (Namkoong *et al.* 1988). Selected elite genotypes obtained from an outbreeding strategy could then be developed into inbred lines as spin-off varieties intended solely for production.

Nonetheless, given regular and consistent selfing, a certain amount of fixation of unfavorable alleles appears to be an inescapable result, which somehow overshadows the possibilities of systematic selfing as a breeding method. We have already pointed at the fact that this fixation results from a trade-off between inbreeding and segregation. Eventual solutions to circumvent this problem could be to combine cycles of selfing and outcrossing, or to use selfing uniquely for the evaluation of parents that could be subsequently selected for outcrossing (backward selection).

### Conclusions

In summary, fixation of unfavorable alleles due to drift and inefficient selection was found to be the main issue for selfing and cross-breeding strategies in tree breeding. For noninbreeding strategies, the ability to prevent fixation of unfavorable alleles and to decrease the effects of ID by slow purging were both found to be important factors for safeguarding short- and long-term genetic gains. In general, strategies devoid of structured restrictions to selection, such as nucleus breeding and breeding within a single population with mass selection, were found to be superior to subline and single breeding population with within-family selection strategies. The inbreeding and cross-breeding strategies of this study could be effective in the first few generations provided that selection was conducted from one single progeny population of high value. Several proposals exist in order to improve inbreeding and cross-breeding, like reducing the fixation of recessive alleles, control of relatedness and selection of superior hybrids. However, among the breeding strategies studied here, nucleus breeding and single breeding population are likely the best long-term choices.

## Supplementary Material

Supporting Information
