# Supplementary material for: Performance of Seven Tree Breeding Strategies Under Conditions of Inbreeding Depression
Source: G3 (Bethesda). 2016 Jan 4;6(3):529–40. doi: 10.1534/g3.115.025767 (PMC4777116; doi:10.1534/g3.115.025767)
Supplement: Supporting Information [file supp_6_3_529__index.html]

Performance of Seven Tree Breeding Strategies Under Conditions of Inbreeding Depression — Supporting Information 

# Performance of Seven Tree Breeding Strategies Under Conditions of Inbreeding Depression

## Supporting Information for Wu, Hallingbäck, and Sánchez, 2016

**Files in this Data Supplement:**

- Figure S1 - Development of the additive genetic variance (*σΑ2*) in breeding population. (.pdf, 498 KB)
- Figure S2 - Development of the inbreeding coefficient in the production population. (.pdf, 282 KB)
- File S1 - Supplementary data information (.docx, 29 KB)
- File S2 - Inbreeding-NUCR. (.txt, 4 KB)
- File S3 - Inbreeding-NUCU. (.txt, 4 KB)
- File S4 - Inbreeding-SBPW. (.txt, 110 KB)
- File S5 - Inbreeding-SELFL. (.txt, 4 KB)
- File S6 - Inbreeding-SELFP. (.txt, 110 KB)
- File S7 - Inbreeding-SUBL. (.txt, 11 KB)
- File S8 - Inbreeding-SBPM. (.txt, 110 KB)
- File S9 - Fortran 95 source code for the version of Metagene suitable for the study of inbreeding strategies and inbreeding depression. (.zip, 72 KB)
