# Supplementary figures and images for: Performance of Seven Tree Breeding Strategies Under Conditions of Inbreeding Depression
Source: G3 (Bethesda). 2016 Jan 4;6(3):529–40. doi: 10.1534/g3.115.025767 (PMC4777116; doi:10.1534/g3.115.025767)

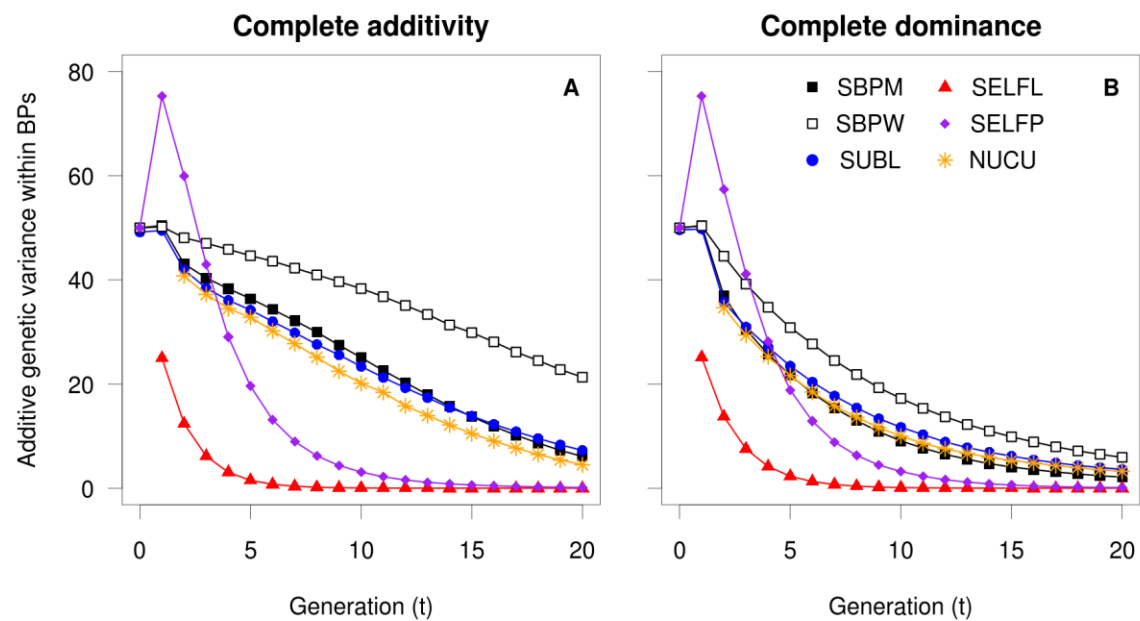

**Figure S1** Development of the additive genetic variance ( $\sigma_A^2$ ) in breeding population.

Supplement: Supporting Information [file supp_g3.115.025767_FigureS1.pdf]

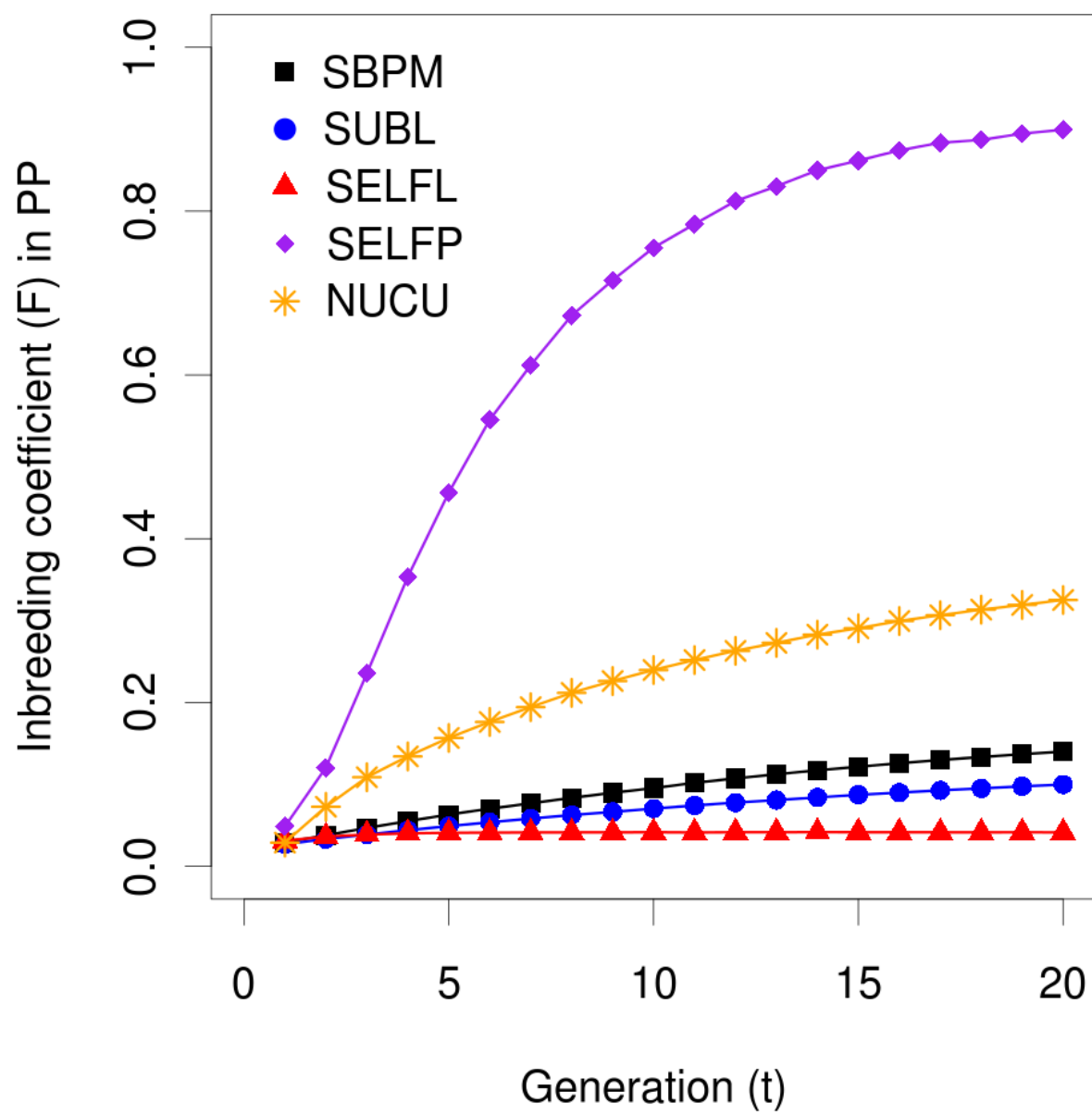

Figure S2 Development of the inbreeding coefficient in the production population.

Supplement: Supporting Information [file supp_g3.115.025767_FigureS2.pdf]
