# Supplementary material for: Performance of Seven Tree Breeding Strategies Under Conditions of Inbreeding Depression
Source: G3 (Bethesda). 2016 Jan 4;6(3):529–40. doi: 10.1534/g3.115.025767 (PMC4777116; doi:10.1534/g3.115.025767)
Supplement: Supporting Information [file supp_g3.115.025767_FileS1.docx]

**Performance of seven tree breeding strategies under conditions of inbreeding depression**

Harry X. Wu*, Henrik R. Hallingbäck*, & Leopoldo Sánchez

**Supplementary data information**

**Recreating breeding strategies and genomic scenario simulations in Metagene**

For this project, the software Metagene version Inb2 was utilized. See Sanchez et al (2007) for further information about the Metagene software.

**Overall workflow for preparing and running simulations**

1. Select the strategy (SBPM, SPBW, SELFP, SUBL, SELFL, SELFI, NUCU or NUCR) to be simulated by selecting one of the txt-type input files. E.g. select Inbreeding-SBPM.txt for the SBPM strategy. The SELFI strategy is the same as SELFL but with four-fold stronger selection intensity (has four times larger segregation populations).
2. Adjust the input file for a specific genomic scenario and save the file using a new name (see details further below).
3. Execution of simulation by running: ./metagenevInb2Lin [input file].txt in Linux environments or: metagenevInb2Win.exe [input file].txt in Windows. The software metagenevInb2Lin and metagenevInb2Win.exe are deposited at http://www.upsc.se/resources/databases-a-software.html and can be downloaded from file folder genomic simulation for execution.
4. See below for types of output files and the name of the parameters given.

**Adjusting the input file for alternative scenarios**

The given input files are designed for a default scenario including 100 completely additive biallelic loci of equal effect (a = 1) and with intermediate allele frequencies (p = 0.5). In order to change to other scenarios you need to adjust the input files.

1. In order to enable the major/minor loci scenario, find the *gene_effects section. This section consists of 100 rows - one for each locus - containing 5 numbers each. The second number in each row signifies the additive effect. For 20 rows (loci), change the second number from 1 to 5. The corresponding numbers at the remainder portion of the rows are left unchanged at 1.
2. In order to change the degree of dominance effect, find the *gene_effects section. The third number in each of the 100 rows signifies the dominance effect for each locus. Change this number for all of the rows from 0 to 0.5 (for partial dominance) or to 1 (for complete dominance). Please note that the effect is absolute, so in case you have major/minor loci scenario you should change the dominance number to 2.5 or to 5 on major effect loci in order to achieve partial and complete dominance respectively. Also note that, for minor/major loci scenarios of the study, only major loci were given dominance effects.
3. In order to enable the U-shaped allele frequency scenario, first change the *Ushape parameter from 0 to -1. Subsequently you copy the 100 frequencies stored in the file Ushape.txt and paste them at the very end of the input txt-file (below the *gene_effects section).

Currently the number of replicates (*n_rep) is set at 500 and such simulation runs will likely require considerable execution time. It is possible to produce results faster (but less reliable) by decreasing the number of replicate simulations. Also note that output filenames can be adjusted by changing the parameters in *fname1, *fname2, *fname5 and *fname6. The output files described by *fname3 and *fname4 are not created unless *n_rep is set at 1 (single replicate run).

**Types of Metagene input, output and process files**

Several input and output files are associated with each Metagene simulation.

These are distinguished by their suffices of the type “.txt”,”.bpa”,

“.ppa”,”.cpa”, “.ped”, “.ppd” and ”.het”.

The different types of files have the following functions.

**TXT.** Is the input text file of simulations and sets important input parameters (dealt with above).

**BPA.** Quantitative genetic parameter output for breeding populations of the simulations (nucleus population in case nucleus breeding is attempted). Several parameters are given as columns in the BPA files (*fname1). Each column is divided in distinct sections for each simulation replicate run. A subheading above each section is given to denote the start of a replicate. Within sections each row constitutes breeding population data for one generation including the zeroth founder generation which is given first within the replicate section. The number of columns and their content depends on the breeding strategy simulated. This type of file has been regularly processed in order to extract key parameters. See further description below for further info.

**CPA.** Quantitative genetic parameter output for the main population in case nucleus breeding is attempted (not available otherwise). Several parameters (in columns) are reported for each generation (in rows) excluding the founder generation. This type of file has been regularly extracted and processed in order to obtain key parameters. See further description below for further info.

**PPA.** Quantitative genetic parameter output for the production population of the simulations. Several parameters (in columns) are reported for each generation (in rows) excluding the founder generation. Replicates are given in row sections. This type of file has been regularly extracted and processed in order to obtain key parameters. See further description below for further info.

**HET.** Locuswise population genetic parameter output for the simulation. A few parameters such as allele frequency and heterozygosity are reported for each locus, population, generation (including the zeroth founder generation) and replicate. Each column is divided in sections for each simulation replicate run. A subheading above each section is given to denote the start of a replicate. Within sections each row constitutes data specific for one locus in the same order as specified in input. The number of columns and their content depends on the breeding strategy simulated. See further description below for further info.

**PED**. Pedigree and genotypic information on each breeding population individual generated in the simulation. In nucleus breeding simulations, individuals in the nucleus are assigned to population 1 while individuals in the main population is assigned to population 2. Is available only for single replicate runs (*n_rep = 1). Data generally not used for the purpose of this study.

**PPD.** Pedigree and genotypic information for each production population individual generated in the simulation. Is available only for single replicate runs (*n_rep = 1). Data generally not used for the purpose of this study.

**Parameter list for BPA-type output files**

*Parameters for each population*

1. MGenotX : Total genotypic population mean for trait X
2. MAdditX : Total additive genetic population mean for trait X
3. MDominX : Total dominance genetic population mean for trait X
4. VGenotX : Genotypic variance in population for trait X
5. VAdditX : Additive genetic variance in population for trait X
6. VAddgenX : Additive genic variance in population for trait X discounting variance due to gametic phase disequilibrium
7. VDomgenX : Dominance genic variance in population for trait X discounting variance due to gametic phase disequilibrium
8. CLdX11 : Gametic phase disequilibrium variance for trait X among loci
9. VPhenX : Phenotypic variance in population for trait X
10. Fiped : Population inbreeding coefficient derived from pedigree data
11. Fimol : Population inbreeding coefficient derived from virtual multiallelic marker data
12. Coaped : Population coancestry coefficient derived from pedigree data
13. Coamol : Population coancestry coefficient derived from virtual multiallelic marker data
14. AlLosX : Number of designated X-alleles lost from the population
15. AlFixX : Number of designated X-alleles fixed in the population

*Metaparameters across populations*

1. Meta_VGenotX : Genotypic variance for trait X across and within populations
2. Meta_VAdditX : Additive genetic variance for trait X across and within populations
3. Meta_VAddgenX : Additive genic variance for trait X across and within populations
4. Meta_VDomgenX : Dominance genic variance for trait X across and within populations
5. Meta_VPhenotX : Phenotypic variance for trait X across and within populations
6. Meta_AlLosX : Number of designated X-alleles lost from all populations
7. Meta_AlFixX : Number of designated X-alleles fixed in all population

The strategy SUBL will show parameters 1-15 for each of the four populations supplying an additional “_pN” after the parameter name for population N. For strategy SELFL, parameters 1-15 are first shown as across line averages (“Mpop_”) and subsequently as across line variances (“Vpop_”). For the NUCR and NUCU strategies, parameters 1-15 describe the nucleus tier (main tier parameters are described in the CPA-files). Parameters 16-22 are given for strategies comprising more than one population (SUBL, NUCR, NUCU, SELFL) and are listed after all other parameters.

**Parameter list for CPA-type output files**

1. MGenotX : Total genotypic population mean for trait X
2. MAdditX : Total additive genetic population mean for trait X
3. MDominX : Total dominance genetic population mean for trait X
4. VGenotX : Genotypic variance in population for trait X
5. VAdditX : Additive genetic variance in population for trait X
6. VAddgenX : Additive genic variance in population for trait X discounting variance due to gametic phase disequilibrium
7. VDomgenX : Dominance genic variance in population for trait X discounting variance due to gametic phase disequilibrium
8. VPhenX : Phenotypic variance in population for trait X
9. Fiped : Population inbreeding coefficient derived from pedigree data
10. Fimol : Population inbreeding coefficient derived from virtual multiallelic marker data
11. Coaped : Population coancestry coefficient derived from pedigree data
12. Coamol : Population coancestry coefficient derived from virtual multiallelic marker data
13. Coaped_tot : Total coancestry (main and nucleus) derived from pedigree data
14. Coamol_tot : Total coancestry (main and nucleus) derived from virtual multiallelic marker data
15. AlLosX : Number of designated X-alleles lost from the population
16. AlFixX : Number of designated X-alleles fixed in the population

The CPA-file only exists for nucleus breeding strategies (NUCU and NUCR) and comprises parameters relevant for the main tier. The only exceptions are parameters 13 and 14 which could be seen as global metaparameters in similarity with parameters 16-22 in the BPA-type files.

**Parameter list for PPA-type output files**

1. MGenotX : Total genotypic population mean for trait X
2. MAdditX : Total additive genetic population mean for trait X
3. MDominX : Total dominance genetic population mean for trait X
4. VGenotX : Genotypic variance in population for trait X
5. VAdditX : Additive genetic variance in population for trait X
6. VAddgenX : Additive genic variance in population for trait X discounting variance due to gametic phase disequilibrium
7. VDomgenX : Dominance genic variance in population for trait X discounting variance due to gametic phase disequilibrium
8. Fiped : Population inbreeding coefficient derived from pedigree data
9. Fimol : Population inbreeding coefficient derived from virtual multiallelic marker data
10. Coaped : Population coancestry coefficient derived from pedigree data
11. Coamol : Population coancestry coefficient derived from virtual multiallelic marker data
12. AlLosX : Number of designated X-alleles lost from the population
13. AlFixX : Number of designated X-alleles fixed in the population

The PPA-file only detail parameters relevant for the production population (PP).

**Parameter list for HET-type output files**

1. AlFr : Locuswise designated allele frequency in breeding population(s)
2. Het : Locuswise heterozygosity in breeding population(s)
3. LocF : Locuswise inbreeding coefficient in breeding population derived by virtual multiallelic markers
4. PrAlFr : Locuswise designated allele frequency in production population
5. PrHet : Locuswise production population heterozygosity
6. BaAlFr : Locuswise designated allele frequency in the main tier
7. BaHet : Locuswise heterozygosity in the main tier.

Columns with these parameters will in general be repeated for each population (p) and generation (t). For example “AlFr_p2t5” describes the allele frequency in population 2 at generation 5. Parameters 1 – 3 are given for each population in SUBL, for average (Mp) and variance (Vp) across populations in the case SELFL and for the nucleus tier in the case of nucleus breeding strategies (NUCU & NUCR). Parameters 4 - 5 describe the production population (PP) and parameters 6 - 7 describes the main tier (if present, e.g. NUCU or NUCR).

**References**

Sanchez L, Yanchuk A A, King J N, 2007, Gametic models for multitrait selection schemes to study variance of response and drift under adverse genetic correlations, Tree Genet. Genomes, 4, 193-200
